# The measure of success: geographic isolation promotes diversification in *Pachydactylus* geckos

**DOI:** 10.1186/s12862-016-0846-2

**Published:** 2017-01-11

**Authors:** Matthew P. Heinicke, Todd R. Jackman, Aaron M. Bauer

**Affiliations:** 1 Department of Natural Sciences, University of Michigan-Dearborn, 4901 Evergreen Rd., Dearborn, MI 48128 USA; 2 Department of Biology, Villanova University, 800 Lancaster Avenue, Villanova, PA 19085 USA

**Keywords:** Biogeography, Systematics, Timetree, Allopatry, Radiation, Cladogenesis, Ancestral reconstruction, Phylogenetic comparative methods

## Abstract

**Background:**

Geckos of the genus *Pachydactylus* and their close relatives comprise the most species-rich clade of lizards in sub-Saharan Africa. Many explanations have been offered to explain species richness patterns of clades. In the *Pachydactylus* group, one possible explanation is a history of diversification via geographic isolation. If geographic isolation has played a key role in facilitating diversification, then we expect species in more species-rich subclades to have smaller ranges than species in less diverse subclades. We also expect traits promoting geographic isolation to be correlated with small geographic ranges. In order to test these expectations, we performed phylogenetic analyses and tested for correlations among body size, habitat choice, range sizes, and diversification rates in the *Pachydactylus* group.

**Results:**

Both body size and habitat use are inferred to have shifted multiple times across the phylogeny of the *Pachydactylus* group, with large size and generalist habitat use being ancestral for the group. Geographic range size is correlated with both of these traits. Small-bodied species have more restricted ranges than large-bodied species, and rock-dwelling species have more restricted ranges than either terrestrial or generalist species. Rock-dwelling and small body size are also associated with higher rates of diversification, and subclades retaining ancestral conditions for these traits are less species rich than subclades in which shifts to small body size and rocky habitat use have occurred. The phylogeny also illustrates inadequacies of the current taxonomy of the group.

**Conclusions:**

The results are consistent with a model in which lineages more likely to become geographically isolated diversify to a greater extent, although some patterns also resemble those expected of an adaptive radiation in which ecological divergence acts as a driver of speciation. Therefore, the *Pachydactylus* group may represent an intermediate between clades in which radiation is adaptive versus those in which it is non-adaptive.

## Background

Discrete geographic regions, both continentally and on islands, often have biotas dominated by a relatively small number of species-rich lineages. The most obvious of these dominant groups are adaptive radiations in which a single ancestral species has given rise to descendants filling numerous niches, examples of which include Galapagos Finches (14 sp., 58% of breeding songbird species in the archipelago), Lake Victoria cichlids (169 sp., 67% of ray-finned fishes in the lake), and West Indies *Anolis* lizards (168 sp., 38% of lizards native to the islands) [1–3]. However, there are many other less-known examples of regionally prominent radiations. Among lizards, one of the most striking are geckos of the genus *Pachydactylus* (56 species) and its close relatives *Chondrodactylus* (6 species), *Colopus* (2 species), and *Elasmodactylus* (2 species). By species number, these geckos are the most successful radiation of lizards in southern Africa. Sixty four of 66 species occur in the southern African subcontinent, defined as that part of Africa south of the Zambezi and Kunene rivers, and most are endemic to this region. These species occupy all major habitat types in southern Africa, and many species in the group display morphological novelties such as loss of adhesive toe pads or the evolution of interdigital webbing [4, 5]. Numerous other gecko genera are found in southern Africa, most of which are endemic or reach their peak diversity there, including *Afroedura*, *Afrogecko*, *Cryptactites*, *Goggia*, *Homopholis*, *Narudasia*, *Ramigekko*, and *Rhoptropus*, but *Pachydactylus* group species often dominate the gekkonid fauna, comprising, for example, 13 of 18 species in the Richtersveld of South Africa [6]. Likewise, none of these other genera approach *Pachydactylus* in its diversity of morphological or ecological variation.

Numerous possible causative factors have been posited or shown to explain the relative success of species-rich organismal groups. In classic adaptive radiations, the rapid evolution of morphological disparity may be the key process in spurring lineage accumulation [7, 8]. In other cases, the evolution of a novel trait may allow organisms possessing that trait to access underutilized resources, with utilization promoting ecological diversification and lineage accumulation. Examples include the evolution of antifreeze proteins in Antarctic icefishes and evolution of the pharyngeal jaw structures in parrotfishes [9, 10]. Sexual selection may also promote lineage accumulation, especially when this selection is for traits that serve as prezygotic isolating mechanisms such as male advertisement calls or color patterns serving as visual mate recognition systems [11, 12]. Finally, in some cases species-rich organismal groups may not actually exhibit a high diversification rate at all, but instead have a longer history of occupancy in a geographic region [13].

In the case of *Pachydactylus* and its relatives, none of these potential explanations are likely to fully account for the observed species diversity. *Pachydactylus* is divided into eight well-defined species groups [14, 15], each of which is believed to be monophyletic, and each of which is morphologically conservative. The degree of morphological disparity between these groups is not of a magnitude expected in a classic adaptive radiation [8]. This is not to say that there is no morphological variation within the genus. Some morphological novelties have evolved, including the previously mentioned foot characteristics as well as variation in scalation. However, such morphological novelties do not appear to be strong drivers of speciation in the group. For example, the most species-rich radiation of *Pachydactylus* geckos that has lost toe pads contains only three species, one of which still has toe pads [4].

Many geckos do have visual display systems or other traits which could theoretically promote diversification via divergent sexual selection. Examples include the semaphore geckos (*Pristurus*), the dwarf geckos (*Sphaerodactylus*), and various day geckos (including *Cnemaspis*, *Lygodactylus*, and *Phelsuma*) [16–18]. In these genera, males are boldly patterned and use signaling behaviors to defend territories or attract mates. *Pachydactylus* and its relatives are strictly nocturnal, however, and typically have a drab pattern. Nor are any other prezygotic isolating mechanisms evident that could plausibly be hypothesized to be under sexual selection. Finally, the relative age of *Pachydactylus* and its relatives likely does not account for its diversity, either – the next closest relative of the *Pachydactylus* group is *Rhoptropus* [5], which also occurs mainly in southern Africa but includes only nine species.

Given that none of these explanations can fully account for the species diversity observed in *Pachydactylus* and its relatives, we hypothesize that geographic isolation leading to allopatric divergence plays a key role in lineage accumulation in *Pachydactylus* and its relatives. Populations in allopatry, if isolated for a sufficient period of time, can naturally speciate via genetic drift without requiring significant contributions from natural selection acting on divergent morphological traits or sexual selection promoting differentiation of mating systems among species [19, 20]. If geographic isolation does play a key role in diversification of *Pachydactylus* and its relatives, then traits promoting the formation of geographic isolation should affect both species’ range sizes and diversification rate. The heritability of range size has been a matter of debate, but increasing numbers of studies demonstrate its heritability [21–25]. In at least some clades, including lizards, this heritability is associated with variable morphological or ecological traits [26, 27]. Likewise, numerous studies have reported trait-associated variation in diversification rates, especially since the development of BiSSE (binary-state speciation and extinction) and related models [28].

For *Pachydactylus* and its relatives, two variable traits of interest that may promote geographic isolation are body size and habitat preference. There is substantial body size variation in *Pachydactylus* and related genera, with the largest and smallest species having adult snout–vent lengths of 35 and 113 mm, respectively [29]. In many groups body size has been shown to be positively correlated with range size [30]. Habitat preference within *Pachydactylus* varies, with species showing preferences ranging from sand dunes to rocky cliffs to houses. In southern Africa, the periodic advance and retreat of Kalahari and Namib sands over geological time is linked to climatic variation [31–33]; this process has likely allowed intermittent connections to form between adjacent rocky habitats, but the prevailing pattern is that terrestrial habitats are relatively continuous while rocky habitats are more discontinuous. As a result, a preference for rocky habitats may be expected to be associated with geographic isolation and smaller range sizes. Such substrate specialization has been suggested to facilitate speciation in the *Pachydactylus* group [14, 34], but has never been explicitly tested.

We test whether body size or habitat preference is associated with the formation of geographic isolation in the *Pachydactylus* group in a phylogenetic context. We have generated a comprehensive time-calibrated multi-locus phylogeny of the group, and obtained body size and habitat preference trait data for all ingroup species. Geographic range size estimates are produced for all species, and the association between trait data and range size is quantified. We also estimate patterns of lineage accumulation through time and trait-associated estimates of diversification. Our data show that both body size and habitat preference affect range size, and that variation in these traits is also correlated with variation in diversification rate, suggesting that allopatric divergence following isolation has played a key role in speciation in the *Pachydactylus* group.

## Methods

### Phylogeny estimation

Previous studies have confirmed that the *Pachydactylus* is part of a monophyletic assemblage of morphologically similar geckos, also including genera *Chondrodactylus*, *Colopus*, and *Elasmodactylus* [4, 14, 35]. We sought to estimate a comprehensive phylogeny for this group, and obtained genetic samples from individuals of 55 of 56 *Pachydactylus* species, 6 of 6 *Chondrodactylus*, both *Colopus* species, and both *Elasmodactylus* species. These genera are part of a larger clade of geckos mainly distributed in Africa and Madagascar, and within this larger grouping they are most closely related to the genus *Rhoptropus* [5, 36]. As such, we included exemplars of 9 of 9 *Rhoptropus* species to serve as a near outgroup. An additional 18 gekkotan and 4 non-gekkotan taxa (*Anolis*, *Gallus*, *Python*, *Trachylepis*) were included as more distant outgroups, with outgroup species choice partially determined based on utility for molecular clock calibration. Nearly all ingroup sequences are associated with vouchered museum specimens. Sequences for four species (*Elasmdactylus tuberculosus*, *Pachydactylus namaquensis*, *P. tsodiloensis*, *P. visseri*) are exceptions, with sequences derived from genetic material obtained from captive-bred individuals; in these cases the live specimens were viewed by the authors to confirm identification and associated genetic material has been deposited in the Cryogenic Collection at the Museum of Comparative Zoology, Harvard University.

We constructed a sequence data set of nuclear and mitochondrial genes that evolve in a relatively clocklike fashion and have proven useful for determining relationships among species within gekkonid genera [37, 38]. The combined data set is 3443 bp (base pairs), including portions of the nuclear genes RAG1 (recombination activating gene 1; 1053 bp), KIF24 (kinesin family member 24; 592 bp) and PDC (phosducin; 395 bp), along with the complete mitochondrial ND2 gene (NADH dehydrogenase subunit 2; 1041 bp) and several adjacent tRNA genes (transfer RNA; 361 bp) (Table 1). All newly generated sequences were deposited in GenBank (accession numbers KY224166–KY224347).

**Table 1:** Specimens and GenBank accession numbers of specimens used in this study

Species	ID Number	ND2	Rag1	PDC	KIF24
*Anolis carolinensis*	n/a	EU747728	AAWZ 02015549	AAWZ 02013979	NW_003338919
*Chondrodactylus angulifer*	MCZ R-184985	KY224209	KY224307	KY224257	
*Chondrodactylus bibronii*	CAS 201841	JN543886	JN543930	KY224258	KY224166
*Chondrodactylus fitzsimonsi*	MCZ R-185712	JN393945	KY224308	KY224259	KY224167
*Chondrodactylus laevigatus*	MCZ R-184819	KY224211	KY224310	KY224260	KY224168
*Chondrodactylus pulitzerae*	CAS 193828	KY224210	KY224309		
*Chondrodactylus turneri*	MCZ R-184410	KY224249	KM073525	KM073612	KM073800
*Coleonyx variegatus*	MVZ 161445	AB114446			
*Coleonyx variegatus*	CAS 205334		EF534777	EF534817	
*Colopus kochii*	CAS 214803	KY224212	KY224311	KY224261	KY224169
*Colopus wahlbergii*	NMZB 16974	JN569158	JN569191	JQ945366	
*Correlophus ciliatus*	AMS R-146595	JX024438	EF534778	EF534818	KU157544
*Elasmodactylus tetensis*	PEM R-5540	KY224213	KY224312	KY224262	KY224170
*Elasmodactylus tuberculosus*	MCZ:Cryo 3006	KY224214	KY224313	KY224263	KY224171
*Euleptes europaea*	no number	JN393941	EF534806	EF534848	KU157420
*Gallus gallus*	n/a	KT626857	NM_001031188	XM_004943303	NC_006127
*Goggia braacki*	PEM R-11911	KM073689	KM073528	KM073614	KM073802
*Nephrurus levis*	AMS 140561		GU459544	GU459746	KU157421
*Nephrurus levis*	SAMA R-19968	AY369018			
*Oedura marmorata*	SAMA R-34209	AY369015			
*Oedura marmorata*	AMS 143861		EF534779	EF534819	KU157428
*Ophidiocephalus taeniatus*	SAM R-44653	AY134601	HQ426303	HQ426214	KU157422
*Pachydactylus acuminatus*	MCZ R-185739	KY224215	KY224314	KY224264	KY224172
*Pachydactylus affinis*	PEM R-17545	KY224216	KY224315	KY224265	
*Pachydactylus amoenus*	AMB 8670	JN569163			
*Pachydactylus angolensis*	CAS 254887	KY224217	KY224316		
*Pachydactylus atorquatus*	MCZ R-184811	KY224218	KY224317	KY224266	
*Pachydactylus austeni*	LSUMZ H1629	KY224250	JQ945321	JQ945389	KY224173
*Pachydactylus barnardi*	MCZ R-184749	KY224219	KY224318	KY224267	
*Pachydactylus bicolor*	NMNW (AMB 7631)	JN543870+ KY224220	JN543911	KY224268	
*Pachydactylus boehmei*	MCZ R-184883	JN543906	JN543947	KY224270	KY224174
*Pachydactylus capensis*	MCZ R-184499	HQ165962	HQ165992	HQ165977	KY224175
*Pachydactylus caraculicus*	MCZ R-185767	JN543889	JN543933	KY224271	
*Pachydactylus carinatus*	LSUMZ 57293	KY224221	KY224319	KY224272	
*Pachydactylus etultra*	MCZ R-184978	HQ165959	HQ165989	HQ165974	KY224176
*Pachydactylus fasciatus*	MCZ R-185759	HQ165949	HQ165978	HQ165963	
*Pachydactylus formosus*	CAS 206715	KY224222	KY224320	KY224273	
*Pachydactylus gaiasensis*	MCZ R-184169	JN543891	KM073533	KM073615	KY224177
*Pachydactylus geitje*	PEM R-11226	JN543887	JN543931	KY224274	KY224178
*Pachydactylus goodi*	MCZ R-184783	KY224223	KY224321	KY224275	KY224179
*Pachydactylus griffini*	MCZ R-185741	KY224224	KY224322	KY224276	KY224180
*Pachydactylus haackei*	CAS 186341	KY224225	KY224323	KY224277	
*Pachydactylus kladaroderma*	PEM R-1253	KY224251	JQ945323	JQ945391	
*Pachydactylus kobosensis*	CAS 223904	KY224226	KY224324	KY224278	KY224181
*Pachydactylus labialis*	MCZ R-184758	KY224227	KY224325	KY224279	KY224182
*Pachydactylus latirostris*	PEM R-16720	JN569141	JN569173	KY224280	KY224183
*Pachydactylus macrolepis*	PEM R-17668	JN569139	JN569170	KY224281	KY224184
*Pachydactylus maculatus*	CAS 186380	KY224228	KY224326	KY224282	KY224185
*Pachydactylus maraisi*	NMNW (JV 1856)	JN543871	JN543912	KY224269	
*Pachydactylus mariquensis*	NMB R10936	JN569157	JN569190	KY224283	KY224186
*Pachydactylus mclachlani*	MCZ R-185094	HQ165950	HQ165980	HQ165965	KY224187
*Pachydactylus monicae*	CAS 193418	HQ165952	HQ165982	HQ165967	KY224188
*Pachydactylus montanus*	MCZ R-184243	KY224229	KY224327	KY224284	KY224189
*Pachydactylus namaquensis*	MBUR 01770	KY224230			
*Pachydactylus namaquensis*	MCZ:Cryo 3007		KY224328	KY224285	KY224190
*Pachydactylus oculatus*	PEM R-1284	KY224231	KY224329	KY224286	
*Pachydactylus oreophilus*	MCZ R-185769	JN543892	JN543936	KY224287	KY224191
*Pachydactylus oshaughnessyi*	NMZB (DGB 611)	KY224232	KY224330	KY224288	KY224192
*Pachydactylus otaviensis*	MCZ R-184867	JN543893	JN543937	KY224289	KY224193
*Pachydactylus parascutatus*	CAS 214750	JN543894	JN543938	KY224290	KY224194
*Pachydactylus punctatus*	PEM R-12461	KY224233	KY224331	KY224291	
*Pachydactylus purcelli*	PEM R-16895	HQ165954	HQ165984	HQ165969	
*Pachydactylus purcelli*	MCZ R-184796				KY224195
*Pachydactylus rangei*	MCZ R-183725	JN543907	JN543948	JQ945392	
*Pachydactylus reconditus*	MCZ R-184856	KY224234	KY224332	KY224292	
*Pachydactylus robertsi*	NMNW R6697	KY224235	KY224333	KY224293	
*Pachydactylus rugosus*	CAS 201905	KY224252	JQ945325	JQ945393	
*Pachydactylus sansteynae*	CAS 214589	JN543898	KY224334	KY224294	
*Pachydactylus scherzi*	MCZ R-184938	KY224236	KY224335	KY224295	
*Pachydactylus scutatus*	MCZ Z37843	JN543901	JN543943	KY224296	KY224196
*Pachydactylus serval*	MCZ R-185989	HQ165956	HQ165986	HQ165986	KY224197
*Pachydactylus tigrinus*	NMB R10936	KY224237	KY224336	KY224297	KY224198
*Pachydactylus tsodiloensis*	MCZ:Cryo 3008	KY224238	KY224337	KY224298	KY224199
*Pachydactylus vansoni*	MCZ R-184434	KY224239		KY224299	KY224200
*Pachydactylus vanzyli*	NMNW (JV1761)	KY224253	JQ945326	JQ945394	KY224201
*Pachydactylus visseri*	MCZ:Cryo 3009	KY224240	KY224338	KY224300	KY224202
*Pachydactylus waterbergensis*	MCZ R-184751	KY224241	KY224339	KY224301	
*Pachydactylus weberi*	PEM R-12449	HQ165960	HQ165990	HQ165975	KY224203
*Pachydactylus werneri*	MCZ R-184960	KY224242	KY224340	KY224302	KY224204
*Phelsuma inexpectata*	JB 56	JN393939	JN393983	JN394016	
*Phelsuma rosagularis*	n/a	EU423292			
*Phelsuma rosagularis*	JB 109		HQ426306	HQ426217	
*Phyllopezus pollicaris*	MZUSP 92491	JX041417	EU293635		
*Phyllopezus pollicaris*	CENPAT12084				JQ827509
*Phyllopezus pollicaris*	JFBM 15822			HQ426225	
*Pygopus nigriceps*	ERP R29509	AY134604			
*Pygopus nigriceps*	SAMA R-23908		FJ571628		
*Pygopus nigriceps*	MVZ 197233			EF534823	
*Python bivittatus*	n/a		AEQU 010344888	AEQU 01027927	NW_006537073
*Python regius*	n/a	AB177878			
*Rhoptropus afer*	MCZ R-183711	KY224254	KM073535	KM073616	KM073806
*Rhoptropus barnardi*	CAS 214658	KY224243	KY224341	KY224303	KY224205
*Rhoptropus benguelensis*	ANG_WC1834	KY224246	KY224346		
*Rhoptropus biporosus*	CAS 224030	KY224244	KY224342	KY224304	KY224206
*Rhoptropus boultoni*	CAS 214713	KY224256	EF534810	EF534852	KY224207
*Rhoptropus bradfieldi*	NMNW (to be accessioned)	KY224245	KY224343	KY224305	
*Rhoptropus diporus*	MCZ R-183737	KY224255	KY224344	KY224306	KY224208
*Rhoptropus montanus*	CAS 254867	KY224247	KY224345		
*Rhoptropus taeniostictus*	CAS 254908	KY224248	KY224347		
*Sphaerodactylus nicholsi*	CAS 198444	KU158020	EF534786	EF534826	KU157415
*Sphaerodactylus roosevelti*	CAS 198428	JN393943	EF534785	EF534825	
*Sphaerodactylus torrei*	JB 34	JX440519	EF534788	EF534829	KU157416
*Teratoscincus microlepis*	JFBM 15				KU157417
*Teratoscincus microlepis*	TG 00074	JX041451	EF534800	EF534842	
*Teratoscincus roborowskii*	CAS 171203	AF114252			
*Teratoscincus roborowskii*	TG 00070		EF534799	EF534841	
*Teratoscincus roborowskii*	JFBM 14				KU157418
*Teratoscincus scincus*	CAS 228808				KU157419
*Teratoscincus scincus*	JFBM 14252	JX041454	EF534801	EF534843	
*Trachylepis varia*	MCZ-R 184873		GU931671		
*Trachylepis varia*	TNHC 68769	GU931603			GU931534
*Trachylepis varia*	ZFMK 68413			KC345241	
*Woodworthia maculata*	RAH 292	GU459852	GU459449	GU459651	
*Woodworthia maculata*	RAH 92				KU157432

Specimen ID codes are as follows: AMB (Aaron M. Bauer field collection); AMS (Australian Museum, Sydney); ANG_WC (Werner Conradie field collection); CAS (California Acedemy of Sciences); CENPAT (Centro Nacional Patagónico, Puerto Madryn); DGB (Donald G. Broadley field collection); ERP (Eric R. Pianka field collection); JB (Jon Boone tissue collection); JFBM (James Ford Bell Museum of Natural History, University of Minnesota); JV (Jens Vindum field collection); LSUMZ (Louisiana State University Museum of Zoology); MBUR (Marius Burger field collection); MCZ (Museum of Comparative Zoology, Harvard University); MVZ (Museum of Vertebrate Zoology, University of California); MZUSP (Museum of Zoology, University of Sao Paulo); NMB (National Museum, Bloemfontein); NMNW (National Museum of Namibia, Windhoek); NMZB (National Museum of Zimbabwe, Bulawayo); PEM (Port Elizabeth Museum); RAH (Rod A. Hitchmough tissue collection); SAM/SAMA (South Australian Museum); TG (Tony Gamble tissue collection); TNHC (Texas Natural History Collection); ZFMK (Zoologisches Forschungsmuseum Alexander Koenig)

For new sequences generated in this study, DNA was obtained from frozen or ethanol-preserved tissue samples using Qiagen DNeasy tissue kits under the manufacturer’s protocol. PCR (polymerase chain reaction) amplification of fragments was performed in 25 μL reactions, under standard reaction conditions [39]. ND2, tRNA, RAG1, and PDC primers used in PCR and sequencing were the same as those used in [37]; KIF24 primers were derived from [40]. PCR purification was performed using AMPure magnetic beads, followed by cycle sequencing and purification using CleanSeq magnetic beads. Capillary electrophoresis was performed on an Applied Biosystems 3730xl sequencer. Sequence assembly was performed using BioEdit [41] or Geneious 5.1 [42], with alignment using Clustal [43]. Alignments of the protein-coding genes were edited manually to preserve reading frame and checked to ensure absence of premature stop codons, while those of the tRNAs were edited manually to preserve secondary structural features estimated in ARWEN [44].

Phylogenetic analyses were performed using maximum likelihood (ML) and Bayesian (BI) optimality criteria. For each analysis, model and partition choices were separately identified under the Bayesian Information Criterion using PartitionFinder [45]. In each case considered models of evolution were limited to those models that can be implemented by the programs used for phylogeny estimation. Greedy search schemes were employed and thirteen potential data blocks were considered: twelve data blocks corresponding to the three codon positions for each of the four protein-coding genes and the tRNA data comprising the thirteenth data block.

The ML analysis was performed using RAxML 8.2.4 [46]. One hundred independent searches were implemented on the original data set to identify the best tree, followed by 1,000 non-parametric bootstrap replicates to assess branch support. Based on the PartitionFinder results, the data were divided into eight partitions, each using one of two models: ND2 codon position 1, ND2 codon position 2, tRNAs, and (PDC position 1 + 2 + RAG1 position 1 + 2) used the GTR (general time reversible) + I + Γ model, while ND2 position 3, (PDC position 3 + RAG1 position 3), (KIF24 position 1 + 2) and KIF24 position 3 used the GTR + Γ model.

The BI analysis was implemented in BEAST 1.8.2 [47], using a Yule tree prior and uncorrelated lognormal relaxed clock. Based on the PartitionFinder results, the data were divided into ten partitions employing six distinct models: ND2 position 1, ND2 position 2, and tRNAs used the GTR + I + Γ model. ND2 position 3 used the GTR + Γ model. RAG1 position 1 + 2 used the TrN (Tamura-Nei) + I + Γ model. RAG1 position 3 and KIF24 position 1 + 2 used the HKY (Hasegawa-Kishino-Yano) + Γ model. PDC position 3 and KIF24 position 3 used the K80 (Kishino 1980) + Γ model. PDC position 1 + 2 used the TrNef + I + Γ model. Four replicate analyses were run for 50 million generations, sampled every 1000 generations. The first 5 million generations were discarded as burn-in. Effective sample sizes were estimated in Tracer 1.5 (>300 for all parameters in each run) to confirm the chain length was adequate.

BEAST 1.8.2 was also used to estimate divergence times simultaneously with phylogenetic relationships. The root prior (Lepidosauria-Archosauria divergence) was given a normal distribution (mean = 275 Ma [million years ago], SD = 15) encompassing the range of estimates for this divergence [48, 49]. Five constraints were also applied to internal nodes: most recent common ancestor (MRCA) of *Phelsuma rosagularis* and *P. inexpectata* (uniform prior; 0–8 Ma; [37]). MRCA of sampled *Sphaerodactylus* – *S. ocoae*, *S. roosevelti*, and *S. torrei* (exponential prior; mean = 3, offset = 15 Ma; [50, 51]). MRCA of *Woodworthia maculata* and *Oedura marmorata* (exponential prior; mean = 17, offset = 16; [52]). MRCA of *Ophidocephalus taeniatus* and *Pygopus nigriceps* (exponential prior; mean = 10, offset = 20; [53, 54]). MRCA of *Teratoscincus roborowskii* and *T. scincus* (exponential prior; mean = 3, offset = 10; [55]).

### Trait data

**Fig. 1:**
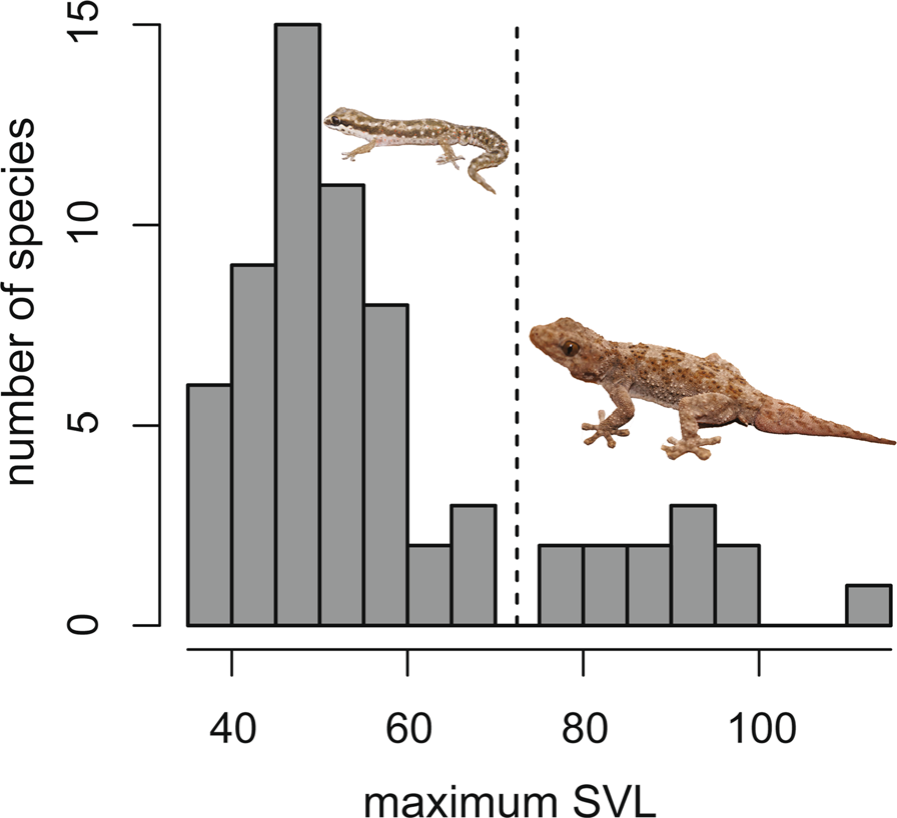
Maximum snout–vent length for species in the genera *Pachydactylus*, *Chondrodactylus*, *Colopus*, and *Elasmodactylus*. Values are based on literature sources along with observations of field-collected and museum-preserved specimens. The small-bodied species *P. geitje* and large-bodied *P. namaquensis* are illustrated

Body size and habitat preference data were assigned to each species based on the authors’ observations of specimens in the wild (62 of 66 ingroup species have been observed in-situ by the authors), supplemented by examination of vouchered museum specimens and information obtained from the literature [29, 56–58]. Maximum body size was treated in two ways depending on analysis. When possible, SVL (snout-vent length) was treated as a continuous character and the log-transformed maximum SVL was used. However, when treatment of size as a continuous character was not computationally feasible we instead treated size as a binary character. In *Pachydactylus* and related genera SVL is bimodal (Fig. 1). Those species with a maximum snout–vent length (SVL) < 70 mm comprised the “small” category, and those with a maximum SVL >75 mm comprised the “large” category. Habitat preference was divided into three categories. Those species that primarily shelter in burrows or under surface debris (logs, loose stones, aloe leaves, etc.), and forage actively on the ground, were classified as “terrestrial.” Species that primarily shelter in rock cracks and forage on cliff faces or boulders were classified as “rupicolous.” Finally, unspecialized species that both shelter and forage on a variety of surfaces (rock faces, tree trunks, buildings, etc.) were classified as “generalist climbers.”

### Range size estimates

Extent of occurrence (EOO) and area of occupancy (AOO) were defined as per the current International Union for the Conservation of Nature (IUCN) standards [1]. EOO was calculated as the area of the minimum convex polygon enclosing distribution records for each respective taxon. AOO was initially calculated as the sum of the total area of the quarter degree grid squares within which at least one record occurs. The final AOO was adjusted to an estimate of the actual suitable habitat within the occupied quarter degree squares based on the literature and the authors’ field knowledge of each species. For all endemic South African species and for most species with a portion of their distribution occurring in South Africa, EOO and AOO values were previously estimated as part of the red list evaluation carried out in association with the Atlas and Red List of the Reptiles of South Africa, Lesotho and Swaziland [58]. Calculated EOO usually provides the broadest possible interpretation of the space used by a species, whereas the AOO represents a quite conservative estimate. However, for taxa known from single localities or several localities that are very close to one another, AOO as calculated above may yield a greater area than EOO. We used EOO or AOO, which ever was the greater, as our estimates of species’ ranges. These values were log transformed when used in analyses.

### Phylogenetic comparative methods

We performed a variety of comparative analyses to investigate the relationship among phylogeny, divergence times, trait data, and range sizes. All comparative analyses were completed in replicate on both the BEAST maximum clade-credibility tree and on 1000 post-burnin trees randomly sampled from the BEAST posterior distribution. These trees were pruned to remove outgroups (for which we have incomplete taxon sampling and no trait data). The package Phytools [59], implemented in R 3.2.2 [60] was used compute phylogenetic signal of range size using both the *K* and *λ* statistics [61, 62]. We also used phytools to plot lineages through time and test for constancy of lineage accumulation through time using the γ statistic of Pybus and Harvey [63].

State dependent diversification of range size on trait data (SVL or habitat) were tested using OUwie [64], which allows for tests of correlation between a multistate vs. continuous trait in a phylogenetic context. Because body size evolution and habitat choice may be coupled [65], we also tested for auto-correlation between these two traits, for a total of three analyses: (1) SVL vs. range size, (2) habitat vs. range size, and (3) SVL vs. habitat. SVL was treated as a multistate (binary) trait in test (1), but was treated as a continuous trait in test (3) to facilitate analysis. In all three cases, we tested the hypothesis that the optimum continuous trait value, *θ*, differed depending on the identity of the multistate trait value, i.e. whether large- and small-bodied species differ in range size (test 1), whether species differing in habitat use differ in range size (test 2), or whether species differing in habitat use differ in body size (test 3). We performed these tests by estimating ancestral states of the multistate character on the phylogeny, and then fitting values of *θ* (trait optimum), *α* (pull toward optimum), and *σ* (rate of change of trait) for the continuous trait under two model regimes. The null Brownian Motion (BM) model regime estimated single values of *θ*, *α*, and *σ* that did not depend on the state of the multistate character. This was tested against a more complex Ornstein-Uhlenbeck (OU) model in which there were multiple *θ* parameters, one per multistate character state. We used ΔAICc (corrected Akaike Information Criterion) values to identify which model provided a better fit for the data. All three tests were performed on 1000 trees randomly sampled from the post-burnin BEAST posterior distribution, and on each of these 1000 trees we performed 100 ancestral reconstructions of the multistate trait using stochastic character mapping [66] implemented in phytools, resulting in a total of 100,000 model fits per test, each with a unique combination of phylogeny and ancestral state estimate.

We also estimated trait-associated rates of speciation (*λ*), extinction (*μ*), and transition rate (*q*) under a BiSSE [28] model for SVL data or a multiple-state speciation and extinction (MuSSE) [67] model for habitat data implemented in Diversitree. Because hypothesis testing in a BiSSE or MuSSE framework can have a high Type I error rate [68–70] and low statistical power when data sets contain fewer than several hundred terminal taxa [71], we refrain from explicitly testing the statistical significance of character-associated variation in model parameter estimates. Instead, we fit models in which each trait was given individual *λ*, *μ*, and *q* parameters strictly to determine estimates of these model parameters. Model fitting was performed in an Markov chain Monte Carlo (MCMC) framework with runs lasting 1100 generations and the first 100 discarded as burn-in. These estimates were obtained for each of 1000 trees randomly sampled from the BEAST posterior distribution, resulting in each parameter estimate being obtained from 1,000,000 observations.

## Results

### Phylogeny and divergence times

**Fig. 2:**
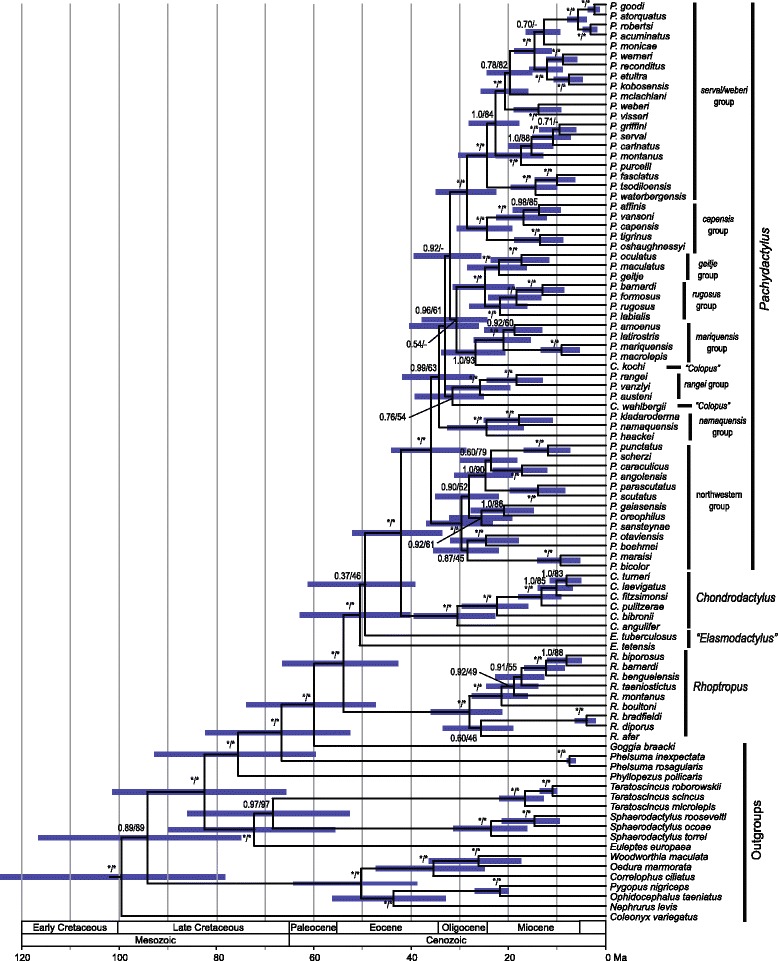
Time-calibrated phylogeny of *Pachydactylus* and related genera. The topology is the maximum clade credibility tree estimated in BEAST with non-gekkotan outgroups cropped for clarity. Support values (Bayesian posterior probabilities/ML bootstrap) are given at nodes; asterisks indicate nodes with Bayesian support values = 1.0 and ML bootstrap values > 95. Named species groups and genera are given to the right. Geologic epochs and eras are indicated on the timescale; post-Miocene epochs (Pliocene, Pleistocene, Holocene) are not labeled

The phylogenies estimated in both the ML and BI analyses are very similar (Fig. 2), and most branches receive strong support. As expected, the grouping of *Pachydactylus*, *Chondrodactylus*, *Colopus*, and *Elasmodactylus* is monophyletic (BI/ML support values 1.0/97) and these are in turn most closely related to *Rhoptropus* (support values 100/1.0). Within the ingroup, the topology resembles that estimated by Bauer and Lamb [14], which included 26 fewer ingroup taxa and was estimated from ~1,600 fewer nucleotide sites, but there are some notable differences. Most notably, both the genera *Colopus* and *Elasmodactylus* are recovered as non-monophyletic. One species of *Colopus*, *C. kochi*, is embedded in *Pachydactylus* and is most closely related to the *Pachydactylus mariquensis* group (support values 1.0/93), a set of four species represented by a single taxon in [14]. The other *Colopus* species, *C. wahlbergii*, is also embedded in *Pachydactylus*, but there is not strong support for any set of *Pachydactylus* species being its closest relatives (support values 0.76/54), although there is strong support for its association with *Pachydactylus* to the exclusion of *Chondrodactylus* and *Elasmodactylus* (100/1.0). The two *Elasmodactylus* species are outside a group containing all *Pachydactylus*, *Colopus*, and *Chondrodactylus* species, with *E. tuberculosus* being more closely related, but with poor support (0.37/46). Within *Pachydactylus*, recognized species groups [14, 15, 35, 72–74] are recovered as monophyletic with strong support as are many of the species-level relationships within these groups. However, within the speciose *serval/weberi* and northwestern groups, in which many new taxa have been added, species relationships are more highly modified. In the first of these, the basal division into reciprocally monophyletic *serval* and *weberi* groups is not supported, and the former makes the latter paraphyletic. Relationships among species groups in *Pachydactylus* remain unresolved, with most groups connected by exceptionally short internodes. There are two exceptions. The *serval/weberi* group and *capensis* group are closest relatives, as are the *geitje* and *rugosus* groups.

**Fig. 3:**
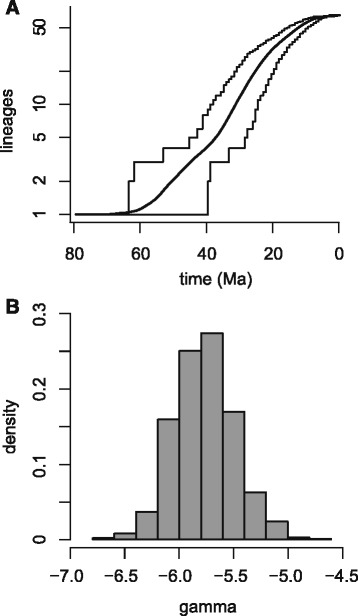
**a** Lineage accumulation in the *Pachydactylus* group. The plot depicts LTT curves for 1000 trees randomly sampled from the BEAST posterior distribution. **b** Histogram of γ statistic estimates for the 1000 LTT curves depicted in (**a**)

The divergences between *Rhoptropus* and *Pachydactylus* + *Chondrodactylus* + *Colopus* + *Elasmodactylus* occurred in the early Cenozoic (66–43 Ma). This is a similar pattern as observed in other gekkonids, in which relatively species-rich regional radiations undergo initial diversification in the early Cenozoic (e.g. [5, 37, 75]). The short internodes connecting *Pachydactylus* species groups are indicative of a relatively high diversification rate in the mid-Cenozoic ~30–35 Ma. The lineage through time (LTT) plot shows that the rate of lineage accumulation remains steady or slowly increases to this point after which there is a noticeable decline (Fig. 3). The overall trend is of significantly decreasing lineage accumulation through time (mean γ value = −5.8, p < 1 x 10^−5^ for all 1000 sampled trees).

### Comparative analyses

**Fig. 4:**
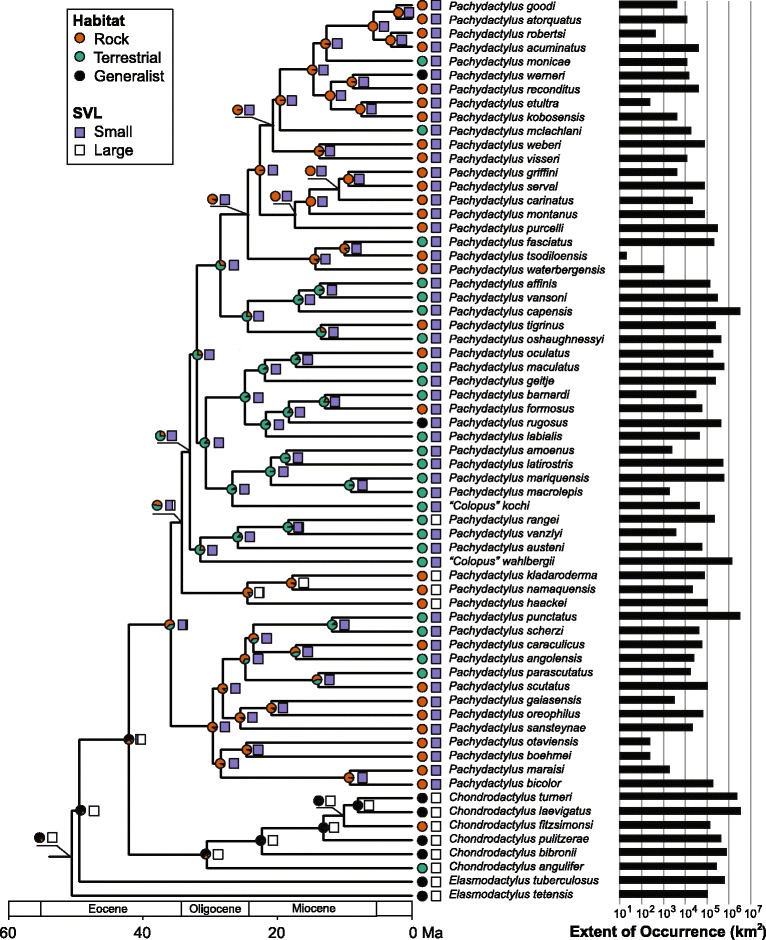
Ancestral states for body size and habitat preference, based on 100 stochastic character maps for each trait on the maximum clade credibility tree. Range size values for each species are given to the right of each terminal branch

**Fig. 5:**
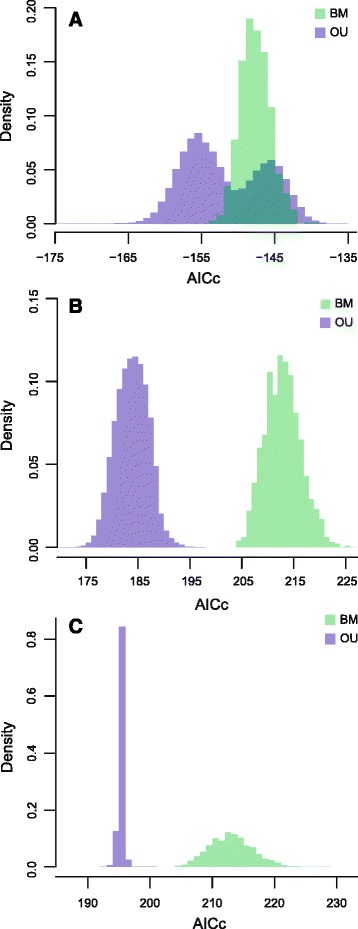
Histogram of AICc values for model fits of Brownian motion (BM) and Ornstein-Uhlenbeck (OU) models of trait diversification estimated in OUwie. **a** habitat preference vs. SVL. **b** habitat preference vs. range size. **c** SVL vs. range size

Ancestral reconstruction of body size in the *Pachydactylus* group suggests that being large-bodied is ancestral for the group (Fig. 4). A shift to small body size occurred once early in the evolutionary history of the group, and there have been only two reversals. Reconstruction of habitat preference is more equivocal, but the common ancestor of the group is most commonly reconstructed as a generalized climber (in 80% of reconstructions). What is clear is that more shifts in habitat preference have occurred than shifts in body size, with approximately 26 transitions indicated in total, most commonly between rock-dwelling and terrestrial habitat preferences. Although both habitat and body size are estimated to have shifted multiple times, including reversals, correlation between the two traits is not particularly strong based on fits of BM and OU models — out of 100,000 model fits, the BM model incorporating only a single global SVL optimum was favored according to the AIC 31% of the time (Fig. 5a).

**Fig. 6:**
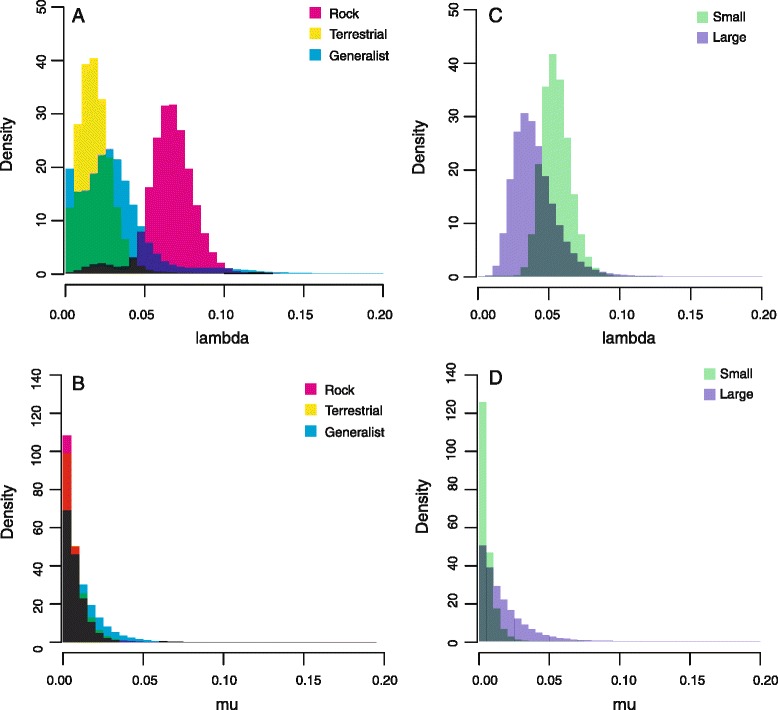
Trait-associated estimates of speciation and extinction generated using Diversitree. Intermediate colors indicate overlap. **a** habitat-associated speciation rate. **b** habitat-associated extinction rate. **c** body size-associated speciation rate. **d** body-size associated extinction rate

In contrast, both body size and habitat preference are strongly correlated with range size (Fig. 5b, c). Range size displays significant phylogenetic signal based on Pagel’s λ (λ = 0.46, p = 0.13), but the estimate of *K* is slightly non-significant (*K* = 0.57, p = 0.077). The estimated global optimum range size (*θ*) for small-bodied species is 10^4.5^ km^2^, approximately one order of magnitude smaller than large-bodied species (*θ* = 10^5.4^ km^2^). When comparing habitat preference, rock-dwelling species have the smallest estimated global optimum extent of occurrence (*θ* = 10^4.1^ km^2^), followed by terrestrial species (*θ* = 10^5.1^ km^2^) with generalized climbers having the largest geographic ranges (*θ* = 10^5.8^ km^2^). Trait-associated estimates of speciation and extinction rates are less variable (Fig. 6). Small-bodied species are estimated to have slightly higher speciation (mean λ[small-bodied] = 0.055; mean λ[large-bodied] = 0.040) and lower extinction rates, but there is extensive overlap. Habitat-associated estimates of diversification rate also overlap, especially between terrestrial species and generalized climbers, although rock-dwelling species are estimated to have speciated at somewhat higher rates (mean λ[generalized climber] = 0.032; mean λ[terrestrial] = 0.012; mean λ[rock-dwelling] = 0.065).

## Discussion

While the heritability of range size has been demonstrated for many lineages, possible mechanistic explanations have varied, and include niche breadth [27], dispersal ability [76, 77], and morphological characteristics [26, 78] of lineages, as well as the geographic limits of biomes, landmasses, or hydrological basins [79]. In many cases, these factors may be interlinked. In this study, we focus on two traits, body size and habitat requirements, that were expected to affect dispersal ability either directly because smaller organisms, including some lizards, may disperse shorter distances [80], or indirectly, because habitat patchiness can restrict dispersal if appropriate dispersal corridors are not available [81]. As expected, within the *Pachydactylus* group the smaller-bodied species occupying more patchily distributed habitats are the species with the smallest geographic ranges. Other studies that have measured dispersal ability directly have shown that reduced dispersal ability does not always lead to reduced range size [77], but in *Pachydactylus* and its relatives our data suggest that dispersal ability and range size are correlated. Traits affecting dispersal ability are likely not the only factors affecting range size, however. Minimally, it is likely that geographic barriers, including major river systems and mountain ranges, also play a significant role in restricting the ranges occupied by individual species. For example, the species *P. austeni* and *P. goodi* are known only from south of the Orange River even though suitable habitats for each of these species also exist to the north [58].

Taken as a whole, the observed patterns of trait evolution, range size, and diversification are consistent with an evolutionary scenario in which diversification has been dominated by geographic isolation followed by allopatric speciation. Based on our analyses, we suggest that geographic isolation has developed more easily in *Pachydactylus* + *Colopus* than it has in *Elasmodactylus* or *Chondrodactylus*, at least partly as a result of *Pachydactylus* + *Colopus* species being more likely to have traits promoting this isolation. Ancestral species in the *Pachydactylus* group as a whole were most likely large-bodied habitat generalists, and most *Chondrodactylus* and *Elasmodactylus* species have retained these traits to the present. We infer small body size and habitat specialization (for either terrestrial or rock-dwelling lifestyles) to appear in the common ancestor of *Pachydactylus* + *Colopus*, coincident with a brief observed increase in the rate of lineage accumulation in the *Pachydactylus* group, followed by a general decline in diversification rate measured across the *Pachydactylus* group as a whole. Rock-dwelling species especially differ strikingly in range size and diversification rate, having extents of occurrence two orders of magnitude smaller than habitat generalists and estimated rates of diversification 2–4X higher than other species. Allopatric speciation of isolated small-bodied, rock-dwelling lineages therefore can account for much of the observed taxonomic diversity in the *Pachydactylus* group. Not surprisingly, the subclades that have retained ancestral traits (*Chondrodactylus* and *Elasmodactylus*) are much less species-rich than those that have not.

The overall decline in diversification rate through time that we observe in the *Pachydactylus* group is similar to patterns documented in many lineages that are often attributed to reduced ecological opportunity through time as niches are filled (e.g. [82–85]). In the case of the *Pachydactylus* group, a general pattern of morphological conservatism within species groups, exemplified by the small number of shifts in body size (Fig. 2) and digital morphology [4, 5] through time is in line with expectations if ecological opportunity has decreased through time. However, shifts in habitat use are more frequent, and the number of co-occurring *Pachydactylus* group species varies from 1 to 13, suggesting that ecological niche space has not been exhausted. An alternative explanation that may also partly explain the observed rate slowdown is a geographic model as described above. In clades dominated by allopatric speciation, diversification rates may decline as vicariance events affect fewer species as species’ geographic ranges decline through time [86, 87]. The relatively low species diversity of *Chondrodactylus*, which includes mostly large-bodied habitat generalists (i.e., species with large geographic ranges), compared to *Pachydactylus*, which includes mostly small-bodied habitat specialists, supports this model.

As indicated above, a jump in lineage accumulation coincident with the appearance of habitat-specialist clades in the mid-Cenozoic ~30–35 Ma is contrary to the general pattern of declining diversification rate through time. It is possible that climatic or geomorphic processes active at this time were especially favorable for isolating lineages, resulting in increased speciation. Major periods of tectonic uplift in eastern and southern Africa did not commence until approximately the Oligocene-Miocene boundary (23 Ma) [88–90], making large-scale geomorphological change incompatible with the observed rate increase. However, a major climatic regime shift did occur at the Eocene-Oligocene boundary: a global cooling associated with Antarctic glaciation [91, 92]. In Africa, this shift resulted in aridification and greater environmental heterogeneity, including reduction in forest cover [93, 94], and would have greatly increased the available habitat for arid-adapted *Pachydactylus* group geckos, potentially facilitating rapid radiation. A similar pattern occurs in forest-adapted chameleons, where rapid radiation is coincident with wide availability of suitable habitat, in the case of chameleons during the Eocene [95].

In performing this study, we have attempted to minimize confounding factors and metholological biases. For example, we collected data for nearly all target taxa and integrated all comparative analyses across a sample of 1000 credible trees to avoid sampling or phylogenetic biases. Even so, our interpretations should be treated cautiously. The observed relationships between trait data and geographic range extent are based on correlation. While we chose to focus on body size and habitat use specifically because we expected them to affect geographic range, it is possible that one or more other factors co-varying with body size and habitat use are the actual drivers of range size variation among species. Trait-associated measurements of diversification rate utilized the BiSSE model. Even though our ingroup phylogeny was comprehensive, the number of taxa in our data set may not have been large enough to avoid inadequacies of the model [69, 71], which is why we refrain from ascribing statistical significance to these results. Alternate methods of trait-dependant diversification (e.g. [70]) likewise are best suited to larger data sets. One possible way to increase data set size is to incorporate taxa from across the Afro-Malagasy clade of geckos, but the interrelationships of genera within this large radiation are still poorly resolved and relevant trait data are missing for many species. Finally, range size estimates are based on known collection localities and a correct interpretation of species-level taxonomy in the group. Collecting effort varies greatly by country, with, for example, less than 10,000 amphibian and reptile collection records in Angola, 38,000 in Namibia, and >100,000 in South Africa [58, 96]. Some species also vary phenotypically and have named subspecies that further study may reveal to warrant specific status (e.g., *Pachydactylus punctatus*; [97]). However, given that trait-associations with range size varied by orders of magnitude, we do not expect refinement of species’ range limits or taxonomy to strongly influence our results.

Beyond interpretation of evolutionary patterns, the results of this study also have significant implications for taxonomy and conservation. The phylogenetic results indicate that *Elasmodactylus* and *Colopus* are not monophyletic, and both species of *Colopus* are nested in *Pachydactylus*. Although we recover *Elasmodactylus* as non-monophyletic, its monophyly cannot be wholly discounted given the poor support for the node joining *E. tuberculosus* with *Chondrodactylus* + *Colopus* + *Pachydactylus*. Performing a Shimodaira-Hasegawa (SH) test also shows that the likelihood of our best-scoring tree (lnL −97035.168404) is not significantly higher than the likelihood of a tree in which *Elasmodactylus* is constrained to be monophyletic (lnL −97036.513963; *p* > 0.05). These species also share morphological traits rare or absent in other *Pachydactylus* group species, including preanal pores and easily broken skin [14]. Thus, we suggest that taxonomic decisions regarding these species be delayed until each species’ phylogenetic position is better established. We refer both *Colopus* species to *Pachydactylus. Colopus wahlbergii* is morphologically divergent and in our analyses its position in *Pachydactylus* is equivocal, with moderate support for an association with the *rangei* group. Thus, we refer it to no species group within *Pachydactylus*. In contrast, *Colophs kochi* is deeply nested in *Pachydactylus*, and there is strong support for its placement as closely related to the *P. mariquensis* group. This species was also included in *Pachydactylus* until recently [14]. We therefore advise that *C. kochi* be re-assigned to the *mariquensis* group within *Pachydactylus*.

One important determinant of rarity is range size, and small range size is a key predictor of extinction risk [98, 99]. Thus, species in the *Pachydactylus* group inheriting traits promoting smaller ranges also inherit traits promoting greater rarity. However, our analyses show these same traits to be associated with higher rates of diversification. Given the difficulty in estimating extinction rates from phylogenies [100], it is unclear if this higher diversification rate is observed despite a higher extinction rate, or if extinction rates do not depend on the measured traits in the *Pachydactylus* group. Notwithstanding this difficulty, these results stress the importance of defining a frame of reference when measuring evolutionary “success.” In the case of the *Pachydactylus* group, more widespread, common species tend to belong to relatively species-poor subclades.

## Conclusions

The relationships among morphological and ecological traits, range size, and diversification that we observe in the *Pachydactylus* group points to a history of geographic isolation contributing significantly to the group’s species richness compared to other African geckos. Even so, some aspects of diversification in the *Pachydactylus* group, including early evolution of divergent traits within the group, are consistent with patterns observed in classic adaptive radiations. In this sense, the process of diversification of *Pachydactylus* group geckos may be considered intermediate between a true adaptive radiation on one hand and a non-adaptive radiation (as observed in plethodontid salamanders; [101, 102]) on the other. It is likely that many other species-rich groups share this same intermediate pattern.

## Abbreviations

AICc: Corrected Akaike information criterion
AOO: Area of occupancy
BI: Bayesian inference
BiSSE: Binary-state speciation and extinction
BM: Brownian motion
bp: Base pairs
EOO: Extent of occurrence
GTR: General time reversible model
HKY: Hasegawa-Kishino-Yano model
IUCN: International union for the conservation of nature
K80: Kimura 1980 model
KIF24: Kinesin family member 24
LTT: Lineages through time
Ma: Million years ago
MCMC: Markov chain Monte Carlo
ML: Maximum likelihood
MRCA: Most recent common ancestor
MuSSE: Multiple-state speciation and extinction
ND2: NADH dehydrogenase subunit 2
OU: Ornstein-Uhlenbeck
PCR: Polymerase chain reaction
PDC: Phosducin
RAG1: Recombination activating gene 1
SH: Shimodaira-Hasegawa
SVL: Snout-vent length
TrN: Tamura-Nei model
tRNA: Transfer RNA
